# MicroRNA discovery by similarity search to a database of RNA-seq profiles

**DOI:** 10.3389/fgene.2013.00133

**Published:** 2013-07-11

**Authors:** Sachin Pundhir, Jan Gorodkin

**Affiliations:** Center for non-coding RNA in Technology and Health, Department of Veterinary Clinical and Animal Sciences (IKVH), University of CopenhagenFrederiksberg C, Denmark

**Keywords:** microRNA, miRNA read profiles, RNA-seq, alignment, deepBlockAlign, read profiles

## Abstract

*In silico* generated search for microRNAs (miRNAs) has been driven by methods compiling structural features of the miRNA precursor hairpin, as well as to some degree combining this with the analysis of RNA-seq profiles for which the miRNA typically leave the drosha/dicer fingerprint of 1–2 ~22 nt blocks of reads corresponding to the mature and star miRNA. In complement to the previous methods, we present a study where we systematically exploit these patterns of *read profiles*. We created two datasets comprised of 2540 and 4795 read profiles obtained after preprocessing short RNA-seq data from miRBase and ENCODE, respectively. Out of 4795 ENCODE read profiles, 1361 are annotated as non-coding RNAs (ncRNAs) and of which 285 are further annotated as miRNAs. Using deepBlockAlign (dba), we align ncRNA read profiles from ENCODE against the miRBase read profiles (cleaned for “self-matches”) and are able to separate ENCODE miRNAs from the other ncRNAs by a Matthews Correlation Coefficient (MCC) of 0.8 and obtain an area under the curve of 0.93. Based on the dba score cut-off of 0.7 at which we observed the maximum MCC of 0.8, we predict 523 novel miRNA candidates. An additional RNA secondary structure analysis reveal that 42 of the candidates overlap with predicted conserved secondary structure. Further analysis reveal that the 523 miRNA candidates are located in genomic regions with MAF block (UCSC) fragmentation and poor sequence conservation, which in part might explain why they have been overlooked in previous efforts. We further analyzed known human and mouse miRNA read profiles and found two distinct classes; the first containing two blocks and the second containing >2 blocks of reads. Also the latter class holds read profiles that have less well defined arrangement of reads in comparison to the former class. On comparison of miRNA read profiles from plants and animals, we observed kingdom specific read profiles that are distinct in terms of both length and distribution of reads within the read profiles to each other. All the data, as well as a server to search miRBase read profiles by uploading a BED file, is available at http://rth.dk/resources/mirdba.

## 1. Introduction

MicroRNAs (miRNAs) are small, non-coding RNAs 18–24 nucleotides in length that play important roles in various biological and metabolic processes, including signal transduction, developmental timing, cell maintenance and differentiation (Zhang et al., 2006b). MiRNAs are involved in post-transcriptional regulation of gene expression by directly cleaving targeted mRNAs or repressing translation (Bartel, 2004). Many *in-vitro* and *in-silico* based approaches have been developed for the prediction of miRNAs. *In-vitro* based approaches like genetic screening approach have contributed to many founding members of miRNAs. However, due to low efficiency and high cost, these are limited for wider applications. Many *in-silico* based approaches have been developed based on major characteristic of miRNAs for example hairpin-shaped stem loop structure integrated with homology search (Wang et al., 2005; Dezulian et al., 2006) or evolutionary conservation (Lai et al., 2003; Lim et al., 2003). Besides, methods based on phylogenetic shadowing (Berezikov et al., 2005), neighbor step loop search (Ohler et al., 2004), minimal folding free energy index (Zhang et al., 2006a) and machine learning approaches have also been developed (Table 1). Various plant and animal miRNAs have been identified using these computational approaches. However, many of these methods have sensitivity problems and give a number of false positive results (Bentwich, 2005). Taken together all search methods aim to reduce the search space in their own respective ways (Lindow and Gorodkin, 2007).

**Table 1:** **Major approaches for the computational prediction of micro-RNA**.

**Approach**	**Programs**	**References**
Evolutionary conservation and stem loop structure	miRseeker and miRscan	Lai et al., 2003; Lim et al., 2003
Neighbor stem loop search	-	Ohler et al., 2004
Sequence based homology and stem loop structure	microHARVESTER, MiRAlign	Wang et al., 2005; Dezulian et al., 2006
Phylogenetic shadowing	-	Berezikov et al., 2005
Minimum free energy index	-	Zhang et al., 2006a
Machine learning methods	ProMiR, mirCoS-a, MiPred	Nam et al., 2005; Jiang et al., 2007; Sheng et al., 2007
RNA-seq based	miRanalyzer, miRDeep2, miRDeep^*^	Hackenberg et al., 2011; Friedländer et al., 2012; An et al., 2013

Recent advances in high throughput sequencing have provided a new opportunity for genome annotation including prediction of novel miRNAs. Many tools like miRDeep2 (Friedländer et al., 2012), miRDeep^*^ (An et al., 2013), and miRanalyzer (Hackenberg et al., 2011) exploit the aggregated set of RNA-seq reads along with secondary structure potential to annotate a genomic locus as miRNA. Indeed, these tools have great ability to predict novel miRNA genes (Williamson et al., 2013). However, these tools do not completely exploit the subtle differences in the arrangement of reads mapped to pre-miRNA. Furthermore, many of these tools start by identifying potential precursor locus for miRNA based on strict rules like fixed precursor size of 110 bp or loop region of size 15 bp. This may result in unconventional miRNA patterns like miRNA-offset RNAs (moRs) that encode for up to four distinct, stable small RNAs (Shi et al., 2009) or plant miRNAs that have different biogenesis mechanism be readily missed by these tools (Lelandais-Briere et al., 2010). Furthermore, many microRNA-sized small RNAs have also been reported to be commonly produced not only from miRNA precursors but also from most other classes of structured RNAs like snoRNA and tRNA (Kawaji et al., 2008; Taft et al., 2009).

**Figure 1:**
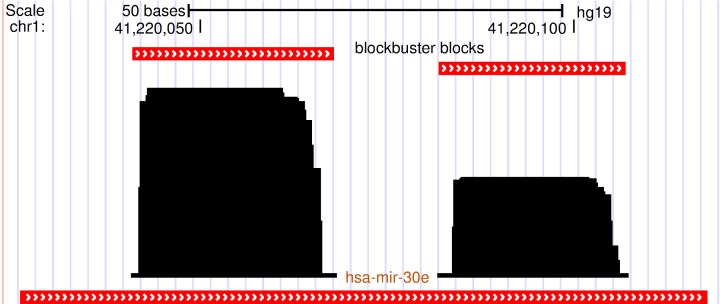
**A characteristic read profile (block group) for microRNA in the human genome**. It is dominated by two distinct clusters of reads (blocks) with almost similar start and/or end positions. These read profiles are in many cases influenced by secondary structures of the parent transcript and may convey information about the processing mechanism of the transcript like dicer cleavage in this case.

**Figure 2:**
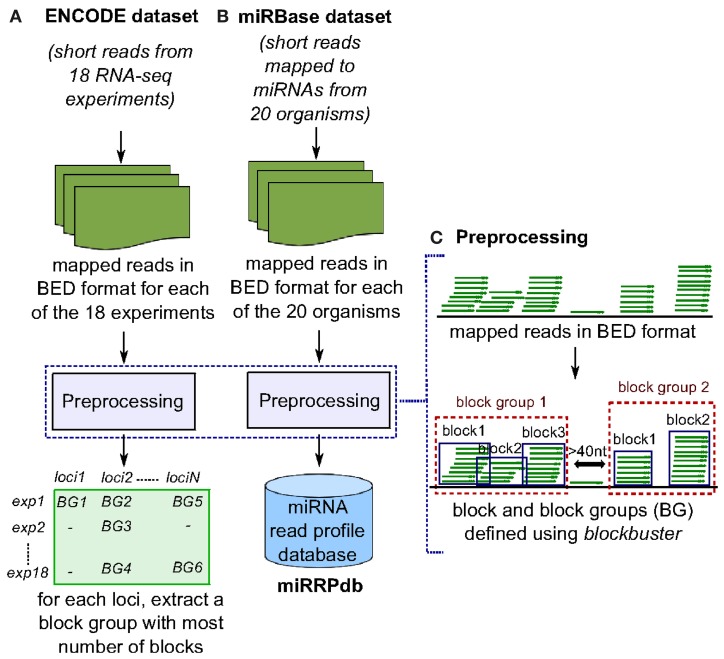
**Preprocessing of ENCODE and miRBase datasets. (A)** In ENCODE dataset, reads mapped to human genome from each of the 18 RNA-seq experiments were subjected to preprocessing to obtain closely spaced set of reads termed here as “block group”. Block groups thus obtained were compiled so as to identify a set of distinct genomic loci that have a block group in at least one experiment. Next, for each locus, we retrieved one block group corresponding to the experiment in which the block group has maximum number of blocks leaving us with 58,161 block groups. **(B)** In miRBase dataset, reads mapped to microRNAs from each of the 20 organisms were subjected to preprocessing and block groups thus obtained were compiled as miRNA read profile database (miRRPdb). **(C)** Given a set of mapped reads in BED format, we derive closely spaced stack of reads termed here as “block group” using blockbuster (Langenberger et al., 2009). Each block group or read profile is composed of one or more blocks of reads.

Several recent studies have recognized that short RNA-seq data, when mapped back to the host genome form read coverage patterns that are distinct and can be used to distinguish between major non-coding RNAs (ncRNAs) such as miRNA, snoRNA and tRNA (Erhard and Zimmer, 2010; Jung et al., 2010; Langenberger et al., 2010, 2012). These read coverage patterns have been referred to as “read profile” or *block group* (see also Figure 2) and are composed of distinctive clusters of reads (blocks) with similar start and/or stop position. The read profiles are often influenced by chemical modifications like in the case of tRNAs (Findeiß et al., 2011), or by secondary structures like in the case of miRNAs where miR and miR^*^ products mutually position with a 3′-overhang that is characteristic for dicer cleavage (Figure 1).

In this study, we present a novel strategy based on pairwise alignment of two read profiles, deepBlockAlign (Langenberger et al., 2012) to predict putative miRNAs in the human genome. We apply this approach on an extensive dataset of read profiles derived from 18 short RNA-seq experiments from ENCODE (ENCODE Consortium, 2011, 2012), and present some putative miRNAs that showed significant similarity to read profiles of known miRNAs from miRBase. We also show distinct classes of miRNA read profiles identified through alignment and hierarchical clustering of read profiles from human and mouse. Furthermore, we present miRNA read profiles that are specific to animals and plants.

## 2. Materials and methods

### 2.1. Datasets

We downloaded two RNA-seq datasets for the analysis of read profiles. First dataset is comprised of short-reads mapped to the human (hg19, Feb. 2009) genome assembly and is downloaded in BAM format from the ENCODE database (ENCODE Consortium, 2011, 2012). This dataset is comprised of 18 RNA-seq experiments performed on nine human tissues with each having two biological replicates. It is to be noted that prior to sequencing, these biological replicates have been grown and isolated independently. In the following, we will refer to this dataset as ENCODE dataset (Table 2). Second dataset is comprised of short-reads mapped to 4862 distinct miRNAs from 20 organisms in miRBase (Kozomara and Griffiths-Jones, 2011). The short reads are derived from 244 GEO experiments performed on various tissues. In the following, we will refer to this dataset as miRBase dataset (Table 3).

**Table 2:** **The ENCODE dataset is comprised of short-reads from 18 RNA-seq experiments performed on nine human tissues with each having two replicates**.

**Tis^a^**	**Replicate1**		**Replicate2**	
	**# reads^b^**	**# BGs^c^**	**# reads^b^**	**# BGs^c^**
Bl	49,280,641	16,437	56,439,584	19,609
Br	48,773,897	15,148	48,394,385	13,317
Bt	26,713,326	13,309	40,144,816	13,555
Cx	41,301,918	15,890	40,798,294	15,948
Ep	47,775,551	13,522	44,163,861	10,923
Es	35,965,377	12,692	33,651,242	14,697
Li	31,930,869	6158	33,939,724	10,684
Lu	38,877,787	14,511	43,732,746	15,873
Sn	37,649,014	6370	41,022,882	11,268

The dataset was downloaded from ENCODE (ENCODE Consortium, 2011) and is composed of a uniform read length of 36 nt.

a Tissues with each having two biological replicates (Bl, Blood; Br, Brain; Bt, Breast; Cx, Cervix; Ep, Epithelium; Es, Embryonic stem cell; Li, Liver; Lu, Lung; Sn, Skin).

b Number of mapped reads.

c Total number of block groups retrieved after preprocessing.

**Table 3:** **miRBase dataset is comprised of short-reads mapped to 4862 distinct microRNAs from 20 organisms**.

**Org^a^**	**# reads^b^**	**# miR^c^**	**# block groups**	
			**All^d^**	**Filter^e^**
Ame	475,288 (1)	159	109	96
Ath	472,5021 (8)	275	303	173
Bfl	37,217 (1)	113	71	34
Bmo	2,021,309 (3)	384	264	194
Cbr	17,442 (1)	115	81	25
Cel	1,048,509 (6)	184	130	93
Cqu	379,978 (1)	68	61	29
Cre	1082 (1)	28	19	11
Crm	9988 (1)	95	63	19
Cte	50,659 (1)	118	72	8
Dme	35,664,132 (50)	237	48	45
Hsa	81,138,802 (79)	1279	801	550
Mmu	913,716,590 (82)	749	688	624
Nve	2711 (1)	34	17	2
Osa	1,506,288 (4)	440	540	288
Ppc	11,176 (1)	113	66	5
Ppt	503,573 (3)	4224	285	148
Rco	147 (25)	13	2	0
Spu	6458 (1)	38	25	7
Tca	4,861,929 (2)	196	193	189
Total	1,046,178,299	4862	3838	2540

Dataset was downloaded from miRBase (Kozomara and Griffiths-Jones, 2011).

a Ame, A. mellifera; Ath, A. thaliana; Bfl, B. floridae; Bmo, B. mori; Cbr, C. briggsae; Cel, C. elegans; Cqu, C. quinquefasciatus; Cre, C. reinhardtii; Crm, C. remanei; Cte, C. teleta; Dme, D. melanogaster; Hsa, H. sapiens; Mmu, M. musculus; Nve, N. vectensis; Osa, O. sativa; Ppc, P. pacificus; Ppt, P. patens; Rco, R. communis; Spu, S. purpuratus; Tca, T. castaneum.

b Number of mapped reads. Total number of GEO experiments are given in brackets. Some experiments are comprised of reads from multiple organisms.

c Total number of distinct miRNAs with mapped reads.

d Total number of block groups or miRNAs retrieved after preprocessing.

e Block groups with >1 block and ≤200 nt in length that are compiled to form a database of miRNA read profiles (miRRPdb).

### 2.2. Preprocessing of dataset

Both datasets were subjected to two pre-processing steps. Firstly, the mapped reads were formatted into BED format. The formatting was done for each of the 18 experiments from ENCODE and 20 organisms from the miRBase dataset, separately (Figures 2A,B). Secondly, each BED format file was processed to identify distinct accumulation of reads by assigning two reads to the same locus, when they were separated by less than 40 nt. We chose 40 nt as the threshold to consider two reads from separate loci based on two observations (a) most short reads are less than 40 nt in length and two genomic loci separated by a region of >40 nt with no mapped reads can most likely be considered as distinct. (b) the loop region of most pre-miRNAs is <40 nt in length. Consecutive reads within a locus were divided into blocks using blockbuster (with parameters: -distance 40, -minBlockHeight 2, -minClusterHeight 10, -scale 0.5) (Langenberger et al., 2009) (Figure 2C). blockbuster merges mapped reads into blocks based on their location in the reference genome. Thus, stacks of reads are combined to read blocks which is analogous to tags (set of reads) processed from a specific locus. This strategy greatly reduces the size of the data set and allows the application of more costly algorithms while maintaining structural properties such as position, length and approximate read start sites and ends. The obtained set of one or more blocks at a locus are then called block groups (Table 2). It is to be noted that for the ENCODE dataset, we discarded all blocks that had read count of <10% with respect to the total reads within its block group in order to (a) ensure all blocks are represented by at least one read, since minimum number of reads in a block group is set to 10 (-minClusterHeight 10); and, (b) nullify the effect of sequencing depth during comparison of block groups across 18 RNA-seq experiments from the ENCODE dataset (Figure 2A). Here after, we will use the term *block group* and *read profile*, synonymously.

Of the 3838 block groups obtained after the preprocessing of miRBase dataset, we filtered 2540 block groups that had more than one block and were ≤200 nt in length. The 2540 block groups were then compiled to from a database of miRNA read profiles (Figure 2B and Table 3) abbreviated hereafter as miRRPdb. Next, for the ENCODE dataset, we derived 58,161 genomic loci where a block group or read profile is observed in at least one tissue (Figure 2A). For each genomic locus, we retrieved one block group corresponding to the tissue in which the block group had the maximum number of blocks leaving us with 58,161 block groups. All the block groups were then compared to known annotation [1049 miRNA from miRBase v16 (Kozomara and Griffiths-Jones, 2011), 513 tRNA loci from gtRNAdb (Chan and Lowe, 2009), 402 snoRNA, 1794 scRNA, 2007 snRNA loci and 722 other RNAs from UCSC annotation (Karolchik et al., 2004)]. Block groups were also compared with 8811 ncRNA annotations from Rfam (Gardner et al., 2011). All the block groups whose coordinate overlapped at ≥1 nt with that of known annotations were designated as “annotated” block groups (Table 4). Similarly, block groups were compared with coordinates of exon, intron, 5′ UTR and 3′ UTR region downloaded from UCSC (Karolchik et al., 2004) and were annotated accordingly, if overlapping at >50% else designated as from intergenic region. If a block group overlaps to more than two genomic regions, then the region with maximum overlap is assigned to it. Of the 58,161 block groups, we filtered 4795 block groups that had more than one block and were ≤200 nt in length. Out of 4795 block groups, 1361 were annotated, and the rest 3434 were unannotated, see Table 4. We used the 1361 annotated block groups as benchmark dataset to evaluate the prediction performance of our method.

**Table 4:** **Annotation status of 58,161 block groups obtained after preprocessing of ENCODE dataset and their alignment to miRRPdb**.

**Annotation**	**# block groups**		**# miRRPdb hits(%)^c^**
	**All^a^**	**Filter^b^**	
miRNA	571	285	223 (78)
snoRNA	468	255	3 (1)
tRNA	625	496	7 (1)
snRNA	395	143	6 (4)
scRNA	187	46	3 (7)
others	277	136	8 (6)
unannotated	55,638	3434	523 (15)
Total	58,161	4795	773 (16)

a Block groups obtained after preprocessing and are overlapping to non-coding RNA annotation.

b Block groups with >1 block and ≤200 nt in length.

c Block groups that have significant alignment score (≥0.7) to miRNA read profile database (miRRPdb).

### 2.3. Performance evaluation

The performance of the proposed method for the prediction of miRNA is evaluated based on sensitivity, specificity and MCC that are computed using a confusion matrix (2 × 2 contingency table). The confusion matrix is essentially composed of four components (a) number of miRNA that are correctly predicted as miRNA (True Positive, TP), (b) number of miRNA that are incorrectly predicted as non-miRNA (False Negative, FN), (c) number of non-miRNA that are incorrectly predicted as miRNA (False Positive, FP); and, (d) number of non-miRNA that are correctly predicted as non-miRNA (True Negatives, TN). The sensitivity measures the proportion of true positives (TP) out of total number of positives (miRNAs) in the benchmark dataset (TP/TP + FN). Like-wise, specificity measures the proportion of true negatives (TN) out of total number of negatives (non-miRNAs) in the benchmark dataset [TN/(TN + FP)]. The MCC is a discrete version of Pearson's correlation coefficient and is widely used in machine learning to measure the quality of (two-class) binary classifications. It is computed as

(1) $$\text{MCC}=\frac{\left(\text{TP}\times \text{TN})-(\text{FP}\times \text{FN}\right)}{\sqrt{\left(\text{TP}+\text{FP})(\text{TP}+\text{FN})(\text{TN}+\text{FP})(\text{TN}+\text{FN}\right)}}$$

### 2.4. Prediction of putative miRNA

**Figure 3:**
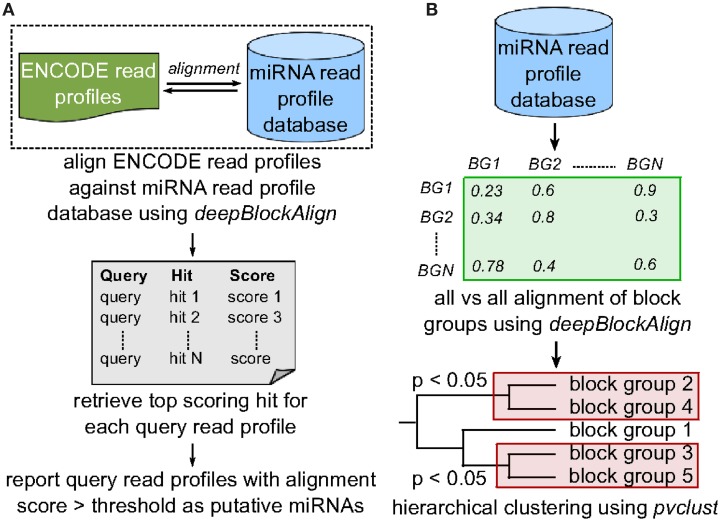
**Search strategy. (A)** To predict putative miRNA candidates, we align 4795 read profiles from ENCODE dataset against miRNA read profile database (miRRPdb) that comprise of 2540 block groups using deepBlockAlign. We retrieve top scoring alignment for each query as putative miRNA, if the score is above the threshold of 0.7. **(B)** To predict distinct classes of miRNA based on their read profiles, all 2540 read profiles from miRRPdb were aligned against each other using deepBlockAlign to generate an alignment score matrix. Next, we perform hierarchical clustering of block groups based on their alignment score using pvclust. pvclust computes the *p*-value for each cluster in hierarchical clustering using multiscale bootstrap resampling and indicates how strong the cluster is supported by the data. We select all the clusters with *p* < 0.05 that represent families of microRNAs that share similar read profiles.

In an earlier study, we have developed a tool named deepBlockAlign for the alignment of two read profiles (Langenberger et al., 2012). deepBlockAlign normalizes the read counts by the total reads within a block group followed by a two-tier strategy to align read profiles. The alignment score from deepBlockAlign ranges from 0, suggesting perfect dissimilarity, to 1 for perfect similarity between the two read profiles. It is to be noted that in the absence of any statistical power or background distribution to evaluate the significance of deepBlockAlign scores (since “background” transcription still has to be defined) (Langenberger et al., 2012), we have used deepBlockAlign to compare the 1361 annotated read profiles from ENCODE dataset against miRRPdb in order to identify a meaningful discriminative dba score (see section 3.1 below). Based on the derived dba score, we align 3434 unannotated read profiles from ENCODE dataset against miRRPdb and identify novel genomic regions that have read profiles similar to those of known miRNAs (Figure 3A).

### 2.5. Prediction of miRNA families

To predict distinct classes of miRNA based on their read profiles, we performed cluster analysis on 550 human and 624 mouse read profiles from miRRPdb, separately. In cluster analysis, we first perform all vs. all alignment of all block groups using deepBlockAlign to generate a square matrix of alignment scores. Second, the R package pvclust (Suzuki and Shimodaira, 2006) is used for hierarchical clustering of block groups based on their alignment scores (Figure 3B). pvclust computes the *p*-value for each cluster in hierarchical clustering using multiscale bootstrap resampling and indicates how strong the cluster is supported by the data. We select all the clusters comprised of at least 15 read profiles at a *p*-value of <0.05, as families of miRNAs that share similar read profiles. To predict any organism specific read profile class, we also performed the cluster analysis on all 2540 read profiles from miRRPdb.

## 3. Results

### 3.1. Benchmarking

**Figure 4:**
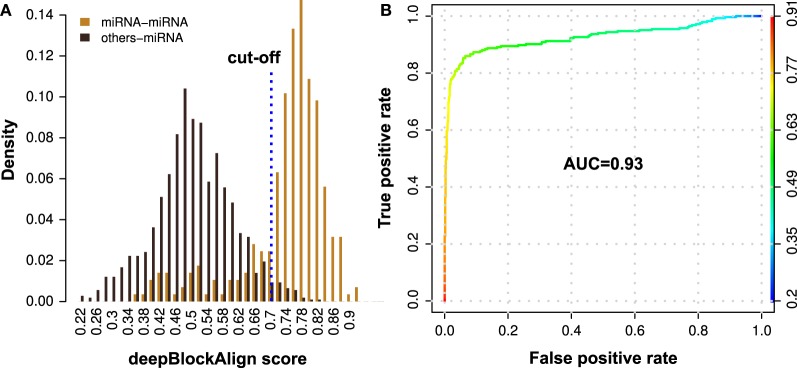
**Performance evaluation of the proposed method for the prediction of putative miRNAs using alignment of read profiles. (A)** Density distribution of deepBlockAlign alignment scores for 285 miRNA (orange) and 1076 other ncRNA (black) read profiles from ENCODE dataset against miRNA read profile database (miRRPdb), respectively. We observed two distinct score distributions comprised of most miRNAs (223 out of 285) that have an alignment score of ≥0.7 and most of the other ncRNAs (1049 out of 1076) with score <0.7 against miRRPdb. **(B)** ROC curve analysis of the prediction performance. A high AUC of 0.93 was observed suggesting that miRNA read profiles have characteristic features that are distinct from read profiles of other ncRNAs and can be employed for confident miRNA prediction.

To benchmark the prediction performance of our proposed method for the prediction of putative miRNAs, we aligned 1361 annotated read profiles (285 miRNA and 1076 other ncRNAs; Table 4) from ENCODE dataset against miRRPdb using deepBlockAlign. For each of the 1361 query read profiles, we selected one read profile from miRRPdb which showed highest alignment score as potential hit for the query read profile. We observed two completely distinct distributions of the alignment scores (Figure 4A), one from alignment of both query and subject read profiles as miRNA (miRNA–miRNA) and another from alignment of any other ncRNA except miRNA as query and miRNA as subject read profile (other-miRNA). ROC curve analysis using R package ROCR (Sing et al., 2005) showed a high AUC of 0.93 suggesting that miRNA read profiles have characteristic features that are distinct from read profiles of other ncRNAs and can be employed for confident prediction of miRNA. (Figure 4B). Indeed, most miRNA–miRNA read profile alignments (223 out of 285) showed an alignment score of ≥0.7 whereas most other-miRNA read profile alignment scores (1049 out of 1076) were <0.7. As the False Positive Rate (FPR) tend to increase above the alignment score of 0.7 (yellow-green intersection) in Figure 4B, we chose 0.7 as the default cut-off alignment score to consider an alignment between unannotated with miRNA read profile as significant and unannotated read profile as putative miRNA candidate.

We further estimated the MCC (Matthews et al., 1975), sensitivity and specificity of the method based on the confusion matrix created for 1361 alignments at a cut-off read profile alignment score of 0.7. The MCC is computed using a confusion matrix that is composed of four components (a) 223 miRNA–miRNA read profile alignments with score ≥0.7 as True Positives (TP), (b) 62 miRNA–miRNA read profile alignments with score <0.7 as False Negatives (FN), (c) 1049 other-miRNA read profile alignments with score <0.7 as True Negatives (TN); and, (d) 27 other-miRNA read profile alignments with score ≥0.7 as False Positives (FP). Based on the confusion matrix, the sensitivity, specificity and MCC of 0.78, 0.97 and 0.80, respectively was observed. We also performed the 5-fold cross validation by splitting the benchmark dataset (1361 annotated read profiles) into five equal and evenly distributed (similar ratio of miRNA and non-miRNA read profiles) datasets. For each of the five rounds of cross-validation, we aligned four of the five datasets against miRRPdb and derived the optimal cut-off of alignment score at which maximum MCC is observed. Using the derived cut-off, the performance of the method is evaluated on the remaining fifth dataset. We observed a mean MCC of 0.80 ± 0.02 and an AUC of 0.93 ± 0.02. A mean cut-off score of 0.7 was observed during the cross-validation and is used hereafter for all the further analysis. In the light of recent reports that many microRNA-sized small RNAs are commonly produced not only from miRNA precursors but also from other classes of structured RNAs like snoRNA and tRNA (Kawaji et al., 2008; Taft et al., 2009), the above measures can be regarded as a reasonable estimate of the performance of this approach in the prediction of novel miRNA candidates.

Furthermore, we compared the performance of our method to an already available tool, miRanalyzer that detect miRNAs using short RNA-seq data (Hackenberg et al., 2011). We chose miRanalyzer because it is one of the widely used tool for miRNA prediction using RNA-seq data, has a prediction performance comparable to other miRNA prediction tools such as miRDeep2 (Williamson et al., 2013) and can be readily applied on our benchmark dataset of mapped reads. We evaluated the performance of miRanalyzer on the mapped reads corresponding to 1361 annotated read profiles from our ENCODE dataset using both the default and model mode of miRanalyzer. In default mode, miRanalyzer made predictions by first mapping reads to known miRNAs from miRBase followed by using random forest model for the remaining set of reads. In model mode, all the predictions are exclusively based on random forest model. We observed an AUC of 0.94 and 0.95 for the former and later mode (Supplementary document available at the web page, http://rth.dk/resources/mirdba). Out of 223 known miRNAs that were correctly predicted by our method, 160 and 208 were also predicted by miRanalyzer using default and model mode. The high performance using the model mode is not surprising since the models have been trained using random forest on the secondary structure features of the same set of known miRNAs within the benchmark dataset. It is to be noted that while computing the AUC, we considered only those ncRNAs, out of 1361 ncRNAs, that have corresponding reads mapped by the miRanalyzer. When computing the AUC for all the 1361 ncRNAs irrespective of their mapping status, we observed a low AUC of 0.64 and 0.68 for the two modes, respectively.

To further evaluate the performance of our method, we analyzed the short RNA-seq data corresponding to Human HeLa cells (GSE10829) that was used to benchmark the performance of miRanalyzer. We observed an AUC of 0.92 for the dataset which is approximately the same AUC of 0.93 observed for 1361 read profiles from the ENCODE dataset. Considering that our method has not implemented any specific miRNA characteristic feature such as hairpin loop size or length of pre-miRNA, it is intriguing that we obtain a high scoring performance of 0.92 in AUC on the original miRanalyzer dataset. In this context, the performance of miRanalyzer on the HeLa dataset has been reported as an AUC of 0.98. Taken together, we observed comparable performance of our method to that of miRanalyzer suggesting that reasonably confident prediction of miRNAs can be performed based on alignment of read profiles. Furthermore, since many of the miRNAs were predicted exclusively by either of the two methods, the diversity in the two prediction approaches can potentially be applied for an enhanced scheme based on the two methods.

### 3.2. Identification of newly added miRNA entries in mirbase

Since, we used miRBase Release 16 to annotate read profiles from the ENCODE dataset, we checked how many of the newly added miRNAs in miRBase Release 19 are identified by our approach. A total of 558 new human miRNAs has been added in the miRBase since release 16. We observed that out of 558, 49 miRNAs are represented as read profiles in our ENCODE dataset. Of these 49 miRNAs, 35 were observed to a have significant alignment score (≥0.7) with miRRPdb, suggesting that the read profile based search can identify most of the newly added miRNAs given that their expression is sufficiently captured during the RNA-seq experiments.

### 3.3. Putative microRNAs

**Figure 5:**
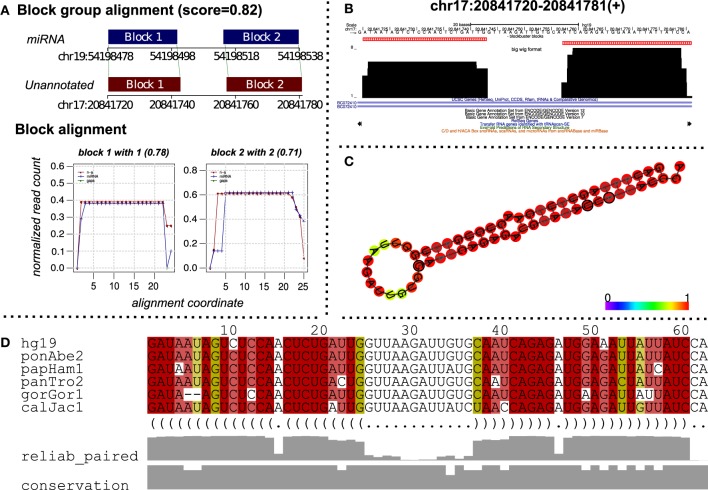
**A putative miRNA predicted by alignment of read profiles. (A)** Alignment between an unannotated and miRNA read profile (block group) computed using deepBlockAlign. The alignment is obtained while aligning unannotated read profiles from ENCODE dataset against the miRRPdb. The unannotated region has a similar read profile to that of miRNA characterized by similar arrangement of reads as evident from block alignment and similar size and distance between the read blocks, leading to a high alignment score of 0.82. **(B)** The read profile for the unannotated region [chr17:20841720–20841781(+)] in UCSC's bigWig format. **(C)** Consensus RNA secondary structure computed using Multiz alignment with high conservation (Blanchette et al., 2004) from six vertebrate genomes corresponding to the unannotated region. The structure has been predicted using PETfold (Seemann et al., 2011) and has a characteristic hair-pin loop structure of miRNAs. **(D)** Multiz alignment (Blanchette et al., 2004) across six vertebrate genomes for the predicted consensus RNA secondary structure.

To predict putative miRNA candidates, we aligned 3434 unannotated read profiles from the ENCODE dataset against miRRPdb using deepBlockAlign. We retrieved 523 alignments with an alignment score of ≥0.7 between an unannotated and miRNA read profile from miRRPdb. Figure 5 shows one such alignment between an unannotated read profile from chr17:20841720-20841781(+) and a read profile of hsa-mir-519b. We can observe high similarity between the two read profiles characterized by similar number, size and distance between the read blocks. Furthermore, the relative arrangement of reads is also very similar between the two, as illustrated through the “Block alignment”. We also checked for the consensus RNA secondary structure by extracting the Multiz alignment (Blanchette et al., 2004) corresponding to the unannotated region from the UCSC browser (Figure 5D). The consensus RNA structure is predicted using the PETfold webserver (Seemann et al., 2011), and it showed a highly conserved hairpin loop structure characteristic for miRNA secondary structure. This further suggests that this region potentially can harbor a miRNA. The unannotated status of this region was checked by searching for possible annotations in miRBase, UCSC tracks of ENCODE/GENCODE (Version 7, 10, and 12), RefSeq, tRNA and snoRNAs.

### 3.4. Putative miRNAs are located in regions that have short or poorly conserved MAF blocks

**Figure 6:**
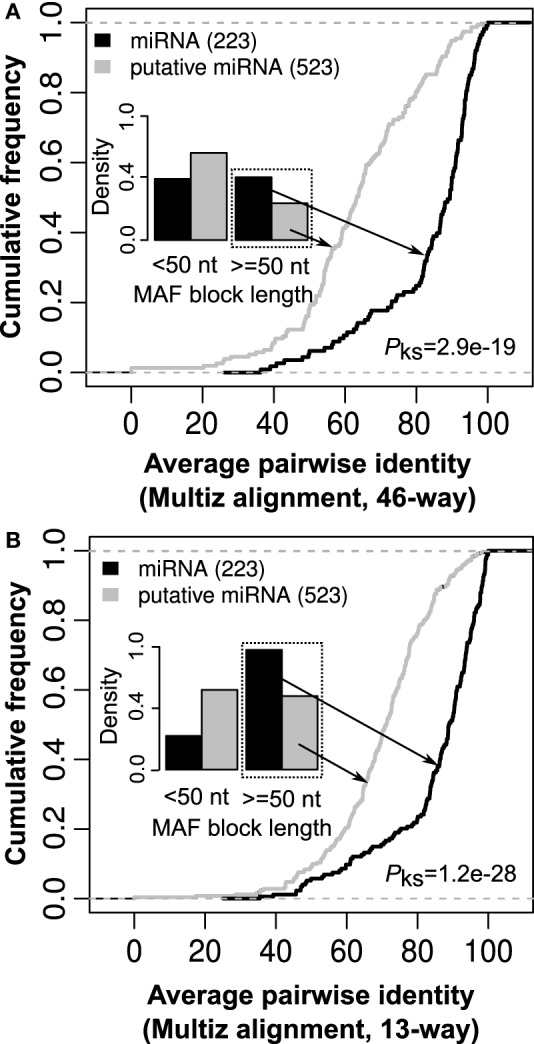
**The length and average pairwise identity for Multiz alignments corresponding to known and putative miRNAs. (A)** In comparison to known miRNAs, most putative miRNAs have Multiz alignment [46-way downloaded from UCSC (Karolchik et al., 2004)] of length <50 nt and rest have a significantly low average pairwise identity (*p*-value = 2.9e-19, Kolmogorov–Smirnov test). **(B)** For the *in house* 13-way Multiz alignments (Anthon et al., in preparation), the same pattern was observed with putative miRNAs either have Multiz alignments that are short (<50 nt) or have a significantly lower average pairwise identity in comparison to known miRNAs (*p*-value = 1.2e-28, Kolmogorov–Smirnov test). This suggests that absence of well-defined Multiz alignments may contribute to many of these putative miRNAs not being identified through methods based on a set of aligned RNA sequences for ncRNA prediction (Gorodkin et al., 2010).

Secondary structure conservation across evolutionary tree is considered a compelling evidence for the biological function of a RNA. Therefore, many tools like Evofold (Pedersen et al., 2006), RNAAlifold (Bernhart et al., 2008), PETfold (Seemann et al., 2008), and RNAz (Gruber et al., 2010) integrate sequence conservation across multiple organisms (Multiz alignments) with RNA folding algorithms for reliable prediction of RNA secondary structure like hairpin loop structure of miRNA. A Multiz alignment represents multiple sequence alignments across a set of species along with measures of evolutionary conservation (Blanchette et al., 2004). For comparative analysis, we retrieved 46-way and 13-way Multiz alignments corresponding to both 523 miRNA candidates and 223 known miRNAs from UCSC (Karolchik et al., 2004) and our *in house* compilation of Multiz alignments, respectively. The *in house* 13-way Multiz alignments have been made on 13 representative organisms in the evolutionary tree (Anthon et al., in preparation). As expected, we observed less MAF block fragmentation over the location of 523 miRNA candidates for the 13-way alignment in comparison to the 46-way alignment, see Figure 6.

For the 13-way alignment, we observed that 272 out of the 523 candidates have too short Multiz alignments (<50 nt) in comparison to 49 out of 223 for known miRNAs (Figure 6). Furthermore, Multiz alignments from putative miRNAs were observed to have a significantly lower average pairwise identity in comparison to that from known miRNAs (Figure 6). A similar pattern of either short or a significantly lower average pairwise identity in Multiz alignments from putative miRNA in comparison to known miRNAs was observed for 46-way Multiz alignments. In this context, absence of well-defined Multiz alignments may well contribute to many of these putative miRNAs not being identified through methods based on a set of aligned RNA sequences for the prediction of non-coding RNAs (Gorodkin et al., 2010).

### 3.5. Secondary structure analysis of putative miRNAs

To independently analyze the 523 miRNA candidate regions for secondary structure, we processed all the corresponding Multiz blocks for quality such as length and average pairwise identity. Interestingly only 278 Multiz alignments have length ≥45 nt and we therefore only carried out prediction on these. Since, miRNAs usually are ~22 nt in length, a Multiz alignment of length <45 can seldom harbor a miRNA hairpin loop structure. Therefore, for the remaining 245 Multiz alignments no attempt to predict RNA secondary structures using sequences from multiple organisms was made. To predict the secondary structure using multiple sequences, we employed two widely used tools, CMfinder (Yao et al., 2006) and RNAz (Gruber et al., 2010), respectively. CMfinder is an expectation maximization algorithm that uses covariance models to predict secondary structure motifs for a set of unaligned sequences (Yao et al., 2006). On the other hand, RNAz is a support vector machine (SVM) based method that evaluates evolutionary conserved pre-aligned set of sequences with thermodynamic stability of RNA secondary structure to detect structural ncRNAs (Gruber et al., 2010). Thus by using these two tools, we aimed to predict secondary structure for regions that are relatively conserved (pre-aligned, RNAz) and regions that are not so well conserved in terms of sequence (unaligned, CMfinder). These methods also complement each other well (Gorodkin et al., 2010).

We used 13-way Multiz alignments corresponding to 278 miRNA candidates as input and considered a *P*-score from CMfinder ≥50 (FDR, False Discovery Rate of 0.27) (Seemann et al., in Preparation) and a *P*-value from RNAz >0.9 (*z*-score < −3 and FDR 0.1) (Gruber et al., 2010) as significant. Based on these thresholds, we predicted conserved RNA secondary structure motifs in 42 putative miRNA candidate regions by CMfinder (17 candidates), RNAz (39 candidates), or by both (14 candidates) CMfinder and RNAz. Out of these 42 putative miRNA candidates, 33 were also predicted as miRNA by miRanalyzer (Hackenberg et al., 2011). Interestingly, for 39 putative miRNA regions predicted to have conserved secondary structure by RNAz, we observed a significantly higher pairwise sequence identity in comparison to rest of the 474 putative miRNA regions (Wilcoxon test, *P*-value < 0.05). This observation again points to the dependency of tools like RNAz on pre-aligned set of sequences for ncRNA prediction as discussed in the previous section. We also observed 13 candidate regions where despite high average pairwise identify of ≥90% in Multiz alignments, no conserved RNA secondary structure was predicted. In conclusion, we obtain 523 novel miRNA candidates of which 42 are further supported by a predicted conserved hairpin loop RNA secondary structure.

### 3.6. Two distinct classes of read profiles in human and mouse

**Figure 7:**
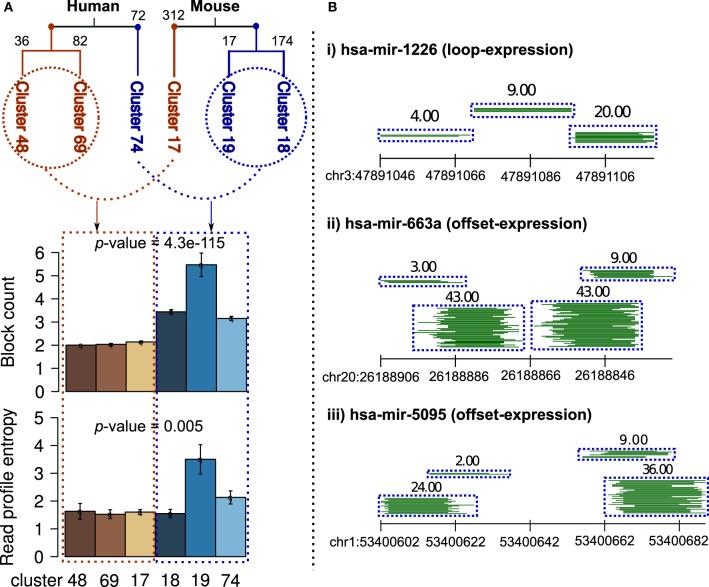
**Clusters of read profiles obtained after hierarchical clustering of 550 and 624 read profiles from Human and Mouse, respectively. (A)** Two distinct and well-separated tree nodes were observed for both plant and animals (red and blue). For better representation, the count of read profiles within each cluster are shown on top of the corresponding branch. In comparison to read profiles in first node, read profiles in second node were characterized by a significantly high number of read blocks (*p*-value = 4.3e-115, Fisher's exact test) and entropy (*p*-value = 0.005, Fisher's exact test, blue shade bars). An earlier study has shown similar finding in *Ciona intestinalis* where half of the miRNA loci encode upto four distinct, stable small RNAs (Shi et al., 2009). **(B)** Three example read profiles with more than two read blocks, (i) beside expression in miR–miR^*^, loop region also showed expression even higher in comparison to miR^*^. (ii and iii) many reads are observed from region partially overlapping to miR–miR^*^, a pattern similar to those of miRNA-offset RNAs (moRs) albeit different in not being completely adjacent to the miR–miR^*^ (Shi et al., 2009).

On independent clustering of 550 and 624 human and mouse known read profiles from miRRPdb, we observed two well-separated tree nodes comprised of three constituent clusters of read profiles for both human and mouse, respectively (Figure 7). In mouse, 503 out of 624 (81%) read profiles were represented in the three clusters. In contrast, only 190 out of 550 (38%) read profiles were represented in the three clusters from human. This might well be due to the sequencing depth in RNA-seq experiments from mouse, which is one order of magnitude higher in comparison to human, see Table 3 for details. Higher sequencing depth helps by including only well-defined read blocks while excluding background read noise during preprocessing step (see section 2) thereby leading to well-defined clusters of read profiles.

Nevertheless, in both human and mouse, we observed two classes of read profiles. The first was comprised of characteristic miRNA-like read profiles which had two read blocks with low entropy (Figure 7A) and the second was comprised of read profiles with a significantly high number of read blocks (*p*-value < 0.05, Fisher's exact test) and entropy (*p*-value < 0.05, Fisher's exact test). Entropy is a measure of the degree of randomness in the arrangement of reads within a read profile (Langenberger et al., 2012 holds the details). An earlier study showed similar findings in *Ciona intestinalis* where half of the miRNA loci encoded upto four distinct, stable small RNAs. The additional RNAs were shown to be generated from sequences immediately adjacent to the predicted ~60 nt pre-miRNA (Shi et al., 2009). Figure 7B illustrates three example of read profiles that have more than two read blocks, first example has expression in loop region that is interestingly even higher in comparison to that of miR^*^ and other two examples with expression from region partially overlapping to miR–miR^*^, a pattern similar to those of miRNA-offset RNAs (moRs) albeit different in not being completely adjacent to the miR–miR^*^ (Shi et al., 2009).

### 3.7. Read profiles specific to plants and animals

**Figure 8:**
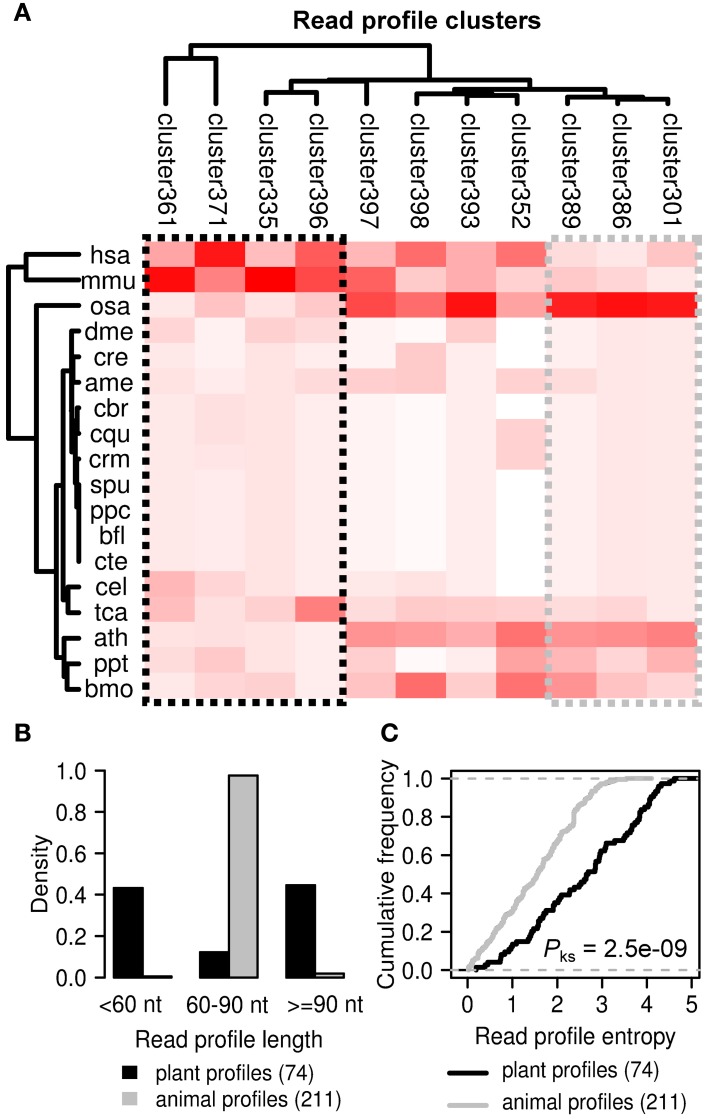
**Distinct clusters of read profiles observed for animals and plants. (A)** Four clusters of read profiles (black box) are mostly observed in *H. sapiens* and *M. musculus* (animal specific). In contrast, three clusters (gray box) are mostly observed in *O. sativa* and *A. thaliana* (plant specific). **(B)** While almost all of the animal specific read profiles are of 60–90 nucleotides in length, plant specific read profiles were either short (<60 nt) or long (≥90 nt) in length. **(C)** Plant specific read profiles were also observed to have a significantly higher entropy in comparison to those from animals (*p*-value < 0.05, Kolmogorov–Smirnov test). This observation may be attributed to the different biogenesis mechanism of miRNAs in plants and animals (Lelandais-Briere et al., 2010).

We also performed alignment and hierarchical clustering of all 2540 read profiles in miRRPdb leaving us with 11 distinct clusters of miRNA read profiles (Figure 8). We observed an interesting pattern of read profile distribution across different organisms, while four cluster of read profiles (cluster 361, 371, 335, and 396) were mostly observed in animals (*hsa, H. sapiens* and *mmu, M. musculus*), three clusters (cluster 389, 386, and 301) were almost exclusively observed in plants (*osa, O. sativa* and *ath, A. thaliana*). The rest of the read profiles clusters were observed across both plant and animals. Next, we compared the length and entropy of the read profiles observed specifically in plant and animals. While almost all of the animal specific read profiles were of 60–90 nucleotides in length, plant specific read profiles were either short (<60 nt) or long (≥90 nt) in length. A higher average length of plant pre-miRNA in comparison to human pre-miRNAs has also been observed in an earlier study (Lindow and Gorodkin, 2007) Plant specific read profiles were also observed to have a significantly higher entropy in comparison to those from animals (*p*-value < 0.05, Kolmogorov–Smirnov test). This observation may be attributed to the different biogenesis mechanism of miRNAs in plants and animals (Lelandais-Briere et al., 2010).

### 3.8. Web server for miRNA identification based on similarity search to miRRPdb

Based on our proposed method, we developed a web server to predict putative miRNA candidates. The web server is available at http://rth.dk/resources/mirdba and facilitate users to align a query read profile against the database of miRNA read profiles (miRRPdb). The standard input is a set of reads mapped to a genomic region of interest in BED format. Users can also adjust several optional parameters that may affect the prediction results, although the default setting is optimal in many cases. The read profile alignment results are presented in a user friendly graphical format and are composed of top two read profiles in miRRPdb that have an alignment score of ≥0.7 (default threshold derived based on benchmarking, see section 3.1) with the query read profile. It should be noted that the alignment score is only an indicator toward similarity of two read profiles. Further checks like similarity in RNA secondary structure should be done to strengthen the prediction results. The alignment of read profiles is visualized using programs developed in PERL, Latex, and R. There is also an option to visualize the read profiles in the UCSC genome browser.

## 4. Discussion

MiRNAs are important regulators of various biological and metabolic processes and computational prediction of miRNA on a genome wide scale is an active research area. We have presented a novel strategy based on alignment of read profiles generated from short RNA-seq data to predict novel miRNA candidates. The alignment of read profiles was performed using a previously published tool, deepBlockAlign. The applicability of the proposed method has been demonstrated by using two short RNA-seq datasets (ENCODE and miRBase datasets). Totals of 4795 and 2540 read profiles were retrieved after preprocessing of ENCODE and miRBase datasets, respectively. 2540 read profiles from miRBase were then compiled to form a database of miRNA read profiles (miRRPdb). Upon alignment of 1361 annotated (285 miRNA and 1024 other ncRNA) read profiles from the ENCODE dataset against miRRPdb, we observed two clearly distinct distributions, one comprised of deepBlockAlign scores between non-miRNA against miRNA read profiles and another comprised of scores between miRNA against miRNA read profiles.

Based on this, we computed the deepBlockAlign score that best separates the two distributions, yielding a classification of 0.8 using MCC with sensitivity, specificity and an area under the curve (ROC) of 0.78, 0.97, and 0.93, respectively. When benchmarking against a representative tool for miRNA prediction based on small RNA-seq data, miRanalyzer (Hackenberg et al., 2011), we observed comparable performance between the two methods on both our dataset as well as the dataset used to benchmark miRanalyzer. Since our method is only based on alignment of read profiles, it has some notable advantages: (a) it can predict putative miRNAs in genomic regions that are devoid of RNA secondary structure information either due to low sequence conservation across multiple organisms that many tools like RNAz (Gruber et al., 2010) require or due to inherent limitation of tools based on single sequence to predict a RNA secondary structure. In this context, many mRNA regions that were predicted to form large, single stranded loops by RNAfold (Gruber et al., 2008) have been shown to form highly base-paired regions using experimental methods (Zheng et al., 2010; Li et al., 2012). (b) it also provides the most similar read profile to a query read profile with respect to arrangement and expression of reads. This can be a useful information, especially when the most similar read profile from miRRPdb is comprised of reads derived from only one experiment, and; (c) similarity search of read profiles can be applied to identify lineage specific miRNA read profiles (see section 3.7) and for the classification of miRNA read profiles based on arrangement of reads (see section 3.6). In total, our method based on alignment of read profiles can be a suitable complement to the other tools such as miRanalyzer and miRDeep that also use short RNA-seq data for miRNA prediction.

Using the proposed method on 3434 uannotated read profiles from ENCODE data set, we predicted 523 unannotated read profiles as putative miRNAs. On RNA secondary structure analysis, 42 of these putative miRNAs were observed to have conserved RNA secondary structure. Furthermore, many of the 523 putative miRNAs were characterized by either short or remarkably low average pairwise identity in corresponding Multiz alignments. Since many RNA secondary structure prediction methods use a set of pre-aligned sequences for ncRNA prediction, absence of well-defined Multiz alignments can contribute to the lack of secondary structure predictions overlapping with many of our miRNA candidates. Global screen of ncRNA candidates using multiple sequence alignment has also been suggested to fail in regions of low sequence similarity (Torarinsson et al., 2006, 2008).

We have shown an unannotated genomic region that share read profile similar to a miRNA along with a characteristic hairpin loop RNA secondary structure with a 3′ overhang. On cluster analysis of read profiles from human and mouse separately, we observed two distinct clusters of read profiles, one with read profiles that have two read blocks and low entropy corresponding to miR–miR^*^ and the other with read profiles that have more than two blocks and a significantly higher entropy in comparison to the former. Many of these additional read blocks were observed in regions adjacent or partially overlapping to miR–miR^*^ and can be possible candidates for miRNA-offset RNAs (moRs).

On cluster analysis of all 2540 read profiles from 18 different organisms in miRRPdb, we observed 11 distinct clusters of read profiles. Interestingly, four of these read profile clusters were mostly observed in animals and another three read profile clusters were mostly observed in plants. The remaining four read profile clusters were observed in both plant and animals. Furthermore, most of the plant specific read profiles were either too

short (<60 nt) or long (≥90 nt) in comparison to animal specific read profiles. Besides, plant read profiles were also observed to have a significantly higher entropy in comparison to the animal read profiles.

Further work includes analysis of miRNA read profiles that have more than two read blocks for potential miRNA-offset miRNAs. In this context, these miRNAs can also be wrongly annotated as miRNA in miRBase. A meta tool integrating the predictions of methods such as miRanalyzer, miRDeep2 and our method would also be useful for the identification of miRNA candidates that are supported by diverse set of prediction approaches. It would also be of wide interest to classify miRNAs based on read profile patterns that are specific to a given tissue, pathological condition or organism. In this context, closer inspection of plant and animal specific read profiles may also reveal novel and organism specific features in their miRNA read profiles.

## Funding

This work is funded in part by the Danish Strategic Research Council (Strategic Growth technologies), the Danish Independent Research Council (Technology and Production), and the Danish Center for Scientific Computation.

### Conflict of interest statement

The authors declare that the research was conducted in the absence of any commercial or financial relationships that could be construed as a potential conflict of interest.

## References

[B1] An J. Lai J. Lehman M. Nelson C. (2013). miRDeep^*^: an integrated application tool for miRNA identification from rna sequencing data. Nucleic Acids Res. 41, 727–737 10.1093/nar/gks1187 23221645 PMC3553977

[B2] Bartel D. (2004). MicroRNAs: genomics, biogenesis, mechanism, and function. Cell 116, 281–297 10.1016/S0092-8674(04)00045-5 14744438

[B3] Bentwich I. (2005). Prediction and validation of microRNAs and their targets. FEBS Lett. 579, 5904–5910 10.1016/j.febslet.2005.09.040 16214134

[B4] Berezikov E. Guryev V. van de Belt J. Wienholds E. Plasterk R. Cuppen E. (2005). Phylogenetic shadowing and computational identification of human microRNA genes. Cell 120, 21–24 10.1016/j.cell.2004.12.031 15652478

[B5] Bernhart S. Hofacker I. Will S. Gruber A. Stadler P. (2008). RNAalifold: improved consensus structure prediction for rna alignments. BMC Bioinform. 9:474 10.1186/1471-2105-9-474 19014431 PMC2621365

[B6] Blanchette M. Kent W. Riemer C. Elnitski L. Smit A. Roskin K. (2004). Aligning multiple genomic sequences with the threaded blockset aligner. Genome. Res. 14, 708–715 10.1101/gr.1933104 15060014 PMC383317

[B7] Chan P. P. Lowe T. M. (2009). GtRNAdb: a database of transfer RNA genes detected in genomic sequence. Nucleic Acids Res. 37, D93–D97 10.1093/nar/gkn787 18984615 PMC2686519

[B8] Dezulian T. Remmert M. Palatnik J. F. Weigel D. Huson D. H. (2006). Identification of plant microRNA homologs. Bioinformatics 22, 359–360 10.1093/bioinformatics/bti802 16317073

[B9] ENCODE Consortium. (2011). A users guide to the encyclopedia of dna elements (encode). PLoS Biol. 9:e1001046 10.1371/journal.pbio.1001046 21526222 PMC3079585

[B10] ENCODE Consortium. (2012). An integrated encyclopedia of DNA elements in the human genome. Nature 489, 57–74 10.1038/nature11247 22955616 PMC3439153

[B11] Erhard F. Zimmer R. (2010). Classification of ncRNAs using position and size information in deep sequencing data. Bioinformatics 26, i426–i432 10.1093/bioinformatics/btq363 20823303 PMC2935403

[B12] Findeiß S. Langenberger D. Stadler P. F. Hoffmann S. (2011). Traces of post-transcriptional RNA modifications in deep sequencing data. Biol. Chem. 392, 305–313 10.1515/bc.2011.043 21345160

[B13] Friedländer M. Mackowiak S. Li N. Chen W. Rajewsky N. (2012). miRDeep2 accurately identifies known and hundreds of novel microRNA genes in seven animal clades. Nucleic Acids Res. 40, 37–52 10.1093/nar/gkr688 21911355 PMC3245920

[B14] Gardner P. Daub J. Tate J. Moore B. Osuch I. Griffiths-Jones S. (2011). Rfam: Wikipedia, clans and the decimal release. Nucleic Acids Res. 39 Suppl. 1, D141 10.1093/nar/gkq1129 21062808 PMC3013711

[B15] Gorodkin J. Hofacker I. L. Torarinsson E. Yao Z. Havgaard J. H. Ruzzo W. L. (2010). *De novo* prediction of structured RNAs from genomic sequences. Trends Biotech. 28, 9–19 10.1016/j.tibtech.2009.09.006 19942311 PMC4712260

[B16] Gruber A. Findeiß S. Washietl S. Hofacker I. Stadler P. (2010). RNAz 2.0: improved noncoding RNA detection, in Pacific Symposium on Biocomputing. (Kamuela, HI: World Scientific), 69–79 19908359

[B17] Gruber A. R. Lorenz R. Bernhart S. H. Neuböck R. Hofacker I. L. (2008). The vienna rna websuite. Nucleic Acids Res. 36 Suppl. 2, W70–W74 10.1093/nar/gkn188 18424795 PMC2447809

[B18] Hackenberg M. Rodríguez-Ezpeleta N. Aransay A. M. (2011). miRanalyzer: an update on the detection and analysis of microRNAs in high-throughput seq-uencing experiments. Nucleic Acids Res. 39 Suppl. 2, W132–W138 10.1093/nar/gkr247 21515631 PMC3125730

[B19] Jiang P. Wu H. Wang W. Ma W. Sun X. Lu Z. (2007). MiPred: classification of real and pseudo microRNA precursors using random forest prediction model with combined features. Nucleic Acids Res. 35 Suppl. 2, W339–W344 10.1093/nar/gkm368 17553836 PMC1933124

[B20] Jung C. H. Hansen M. A. Makunin I. V. Korbie D. Mattick J. (2010). Identification of novel non-coding RNAs using profiles of short sequence reads from next generation sequencing data. BMC Genomics 11:77 10.1186/1471-2164-11-77 20113528 PMC2825236

[B21] Karolchik D. Hinrichs A. S. Furey T. S. Roskin K. M. Sugnet C. W. Haussler D. (2004). The UCSC table browser data retrieval tool. Nucleic Acids Res. 32, D493–D496 10.1093/nar/gkh103 14681465 PMC308837

[B22] Kawaji H. Nakamura M. Takahashi Y. Sandelin A. Katayama S. Fukuda S. (2008). Hidden layers of human small RNAs. BMC Genomics 9:157 10.1186/1471-2164-9-157 18402656 PMC2359750

[B23] Kozomara A. Griffiths-Jones S. (2011). miRBase: integrating microRNA annotation and deep-sequencing data. Nucleic Acids Res. 39, D152–D157 10.1093/nar/gkq1027 21037258 PMC3013655

[B24] Lai E. C. Tomancak P. Williams R. W. Rubin G. M. (2003). Computational identification of drosophila microRNA genes. Genome. Biol. 4:R42 10.1186/gb-2003-4-7-r42 12844358 PMC193629

[B25] Langenberger D. Bermudez-Santana C. Hertel J. Hoffmann S. Khaitovich P. Stadler P. (2009). Evidence for human microRNA-offset RNAs in small RNA sequencing data. Bioinformatics 25, 2298–2301 10.1093/bioinformatics/btp419 19584066

[B26] Langenberger D. Bermudez-Santana C. I. Stadler P. F. Hoffmann S. (2010). Identification and classification of small RNAs in transcriptome sequence data, in Pacific Symposium on Biocomputing (Kamuela, HI), 80–87 10.1142/9789814295291_0010 19908360

[B27] Langenberger D. Pundhir S. Ekstrøm C. Stadler P. Hoffmann S. Gorodkin J. (2012). deepblockalign: a tool for aligning rna-seq profiles of read block patterns. Bioinformatics 28, 17–24 10.1093/bioinformatics/btr598 22053076 PMC3244762

[B28] Lelandais-Briere C. Sorin C. Declerck M. Benslimane A. Crespi M. Hartmann C. (2010). Small RNA diversity in plants and its impact in development. Curr. Genom. 11, 14 10.2174/138920210790217918 20808519 PMC2851111

[B29] Li F. Zheng Q. Ryvkin P. Dragomir I. Desai Y. Aiyer S. (2012). Global analysis of RNA secondary structure in two metazoans. Cell Rep. 1, 69–82 10.1016/j.celrep.2011.10.002 22832108

[B30] Lim L. P. Lau N. C. Weinstein E. G. Abdelhakim A. Yekta S. Rhoades M. W. (2003). The microRNAs of caenorhabditis elegans. Genes Dev. 17, 991–1008 10.1101/gad.1074403 12672692 PMC196042

[B31] Lindow M. Gorodkin J. (2007). Principles and limitations of computational microRNA gene and target finding. DNA Cell Biol. 26, 339–351 10.1089/dna.2006.0551 17504029

[B32] Matthews B. (1975). Comparison of the predicted and observed secondary structure of t4 phage lysozyme. Biochim. Biophys. Acta 405, 442 10.1016/0005-2795(75)90109-9 1180967

[B33] Nam J.-W. Shin K.-R. Han J. Lee Y. Kim V. N. Zhang B.-T. (2005). Human microRNA prediction through a probabilistic co-learning model of sequence and structure. Nucleic Acids Res. 33, 3570–3581 10.1093/nar/gki668 15987789 PMC1159118

[B34] Ohler U. Yekta S. Lim L. P. Bartel D. P. Burge C. B. (2004). Patterns of flanking sequence conservation and a characteristic upstream motif for microRNA gene identification. RNA 10, 1309–1322 10.1261/rna.5206304 15317971 PMC1370619

[B35] Pedersen J. Bejerano G. Siepel A. Rosenbloom K. Lindblad-Toh K. Lander E. (2006). Identification and classification of conserved RNA secondary structures in the human genome. PLoS Comput. Biol. 2:e33 10.1371/journal.pcbi.0020033 16628248 PMC1440920

[B36] Seemann S. Gorodkin J. Backofen R. (2008). Unifying evolutionary and thermodynamic information for RNA folding of multiple alignments. Nucleic Acids Res. 36, 6355–6362 10.1093/nar/gkn544 18836192 PMC2582601

[B37] Seemann S. Menzel P. Backofen R. Gorodkin J. (2011). The PETfold and PETcofold web servers for intra-and intermolecular structures of multiple rna sequences. Nucleic Acids Res. 39 Suppl 2, W107–W111 10.1093/nar/gkr248 21609960 PMC3125731

[B38] Sheng Y. Engström P. G. Lenhard B. (2007). Mammalian microRNA prediction through a support vector machine model of sequence and structure. PLoS ONE 2:e946 10.1371/journal.pone.0000946 17895987 PMC1978525

[B39] Shi W. Hendrix D. Levine M. Haley B. (2009). A distinct class of small RNAs arises from pre-miRNA-proximal regions in a simple chordate. Nat. Struct. Mol. Biol. 16, 183–189 10.1038/nsmb.1536 19151725 PMC2746024

[B40] Sing T. Sander O. Beerenwinkel N. Lengauer T. (2005). Rocr: visualizing classifier performance in r. Bioinformatics 21, 3940–3941 10.1093/bioinformatics/bti623 16096348

[B41] Suzuki R. Shimodaira H. (2006). Pvclust: an R package for assessing the uncertainty in hierarchical clustering. Bioinformatics 22, 1540–1542 10.1093/bioinformatics/btl117 16595560

[B42] Taft R. J. Glazov E. A. Lassmann T. Hayashizaki Y. Carninci P. Mattick J. S. (2009). Small RNAs derived from snoRNAs. RNA 15, 1233–1240 10.1261/rna.1528909 19474147 PMC2704076

[B43] Torarinsson E. Sawera M. Havgaard J. H. Fredholm M. Gorodkin J. (2006). Thousands of corresponding human and mouse genomic regions unalignable in primary sequence contain common RNA structure. Genome. Res. 16, 885–889 10.1101/gr.5226606 16751343 PMC1484455

[B44] Torarinsson E. Yao Z. Wiklund E. Bramsen J. Hansen C. Kjems J. (2008). Comparative genomics beyond sequence-based alignments: RNA structures in the ENCODE regions. Genome. Res. 18, 242–251 10.1101/gr.6887408 18096747 PMC2203622

[B45] Wang X. Zhang J. Li F. Gu J. He T. Zhang X. (2005). MicroRNA identification based on sequence and structure alignment. Bioinformatics 21, 3610–3614 10.1093/bioinformatics/bti562 15994192

[B46] Williamson V. Kim A. Xie B. McMichael G. O. Gao Y. Vladimirov V. (2013). Detecting miRNAs in deep-sequencing data: a software performance comparison and evaluation. Brief. Bioinform. 14, 36–45 10.1093/bib/bbs010 23334922 PMC3999373

[B47] Yao Z. Weinberg Z. Ruzzo W. L. (2006). CMfinder- a covariance model based RNA motif finding algorithm. Bioinformatics 22, 445–452 10.1093/bioinformatics/btk008 16357030

[B48] Zhang B. Pan X. Cox S. Cobb G. Anderson T. (2006a). Evidence that miRNAs are different from other RNAs. Cell. Mol. Life Sci. 63, 246–254 10.1007/s00018-005-5467-7 16395542 PMC11136112

[B49] Zhang B. Pan X. Wang Q. Cobb G. Anderson T. (2006b). Computational identification of microRNAs and their targets. Comput. Biol. Chem. 30, 395–407 10.1016/j.compbiolchem.2006.08.006 17123865

[B50] Zheng Q. Ryvkin P. Li F. Dragomir I. Valladares O. Yang J. (2010). Genome-wide double-stranded rna sequencing reveals the functional significance of base-paired rnas in arabidopsis. PLoS Genet. 6:e1001141 10.1371/journal.pgen.1001141 20941385 PMC2947979

